# Osteogenic Effect of ZnO-Mesoporous Glasses Loaded with Osteostatin

**DOI:** 10.3390/nano8080592

**Published:** 2018-08-04

**Authors:** Rebeca Pérez, Sandra Sanchez-Salcedo, Daniel Lozano, Clara Heras, Pedro Esbrit, María Vallet-Regí, Antonio J. Salinas

**Affiliations:** 1 Departamento de Química en Ciencias Farmacéuticas, Facultad de Farmacia, Universidad Complutense de Madrid, UCM, Instituto de Investigación Hospital 12 de Octubre, imas12, 28040 Madrid, Spain; rebecapr93@gmail.com (R.P.); sansanch@ucm.es (S.S.-S.); danlozan@ucm.es (D.L.); claheras@ucm.es (C.H.); pesbrit@gmail.com (P.E.); vallet@ucm.es (M.V.-R.); 2 Networking Research Center on Bioengineering, Biomaterials and Nanomedicine (CIBER-BBN), 28040 Madrid, Spain; 3 Instituto de Investigación Sanitaria (IIS)-Fundación Jiménez Díaz, 28040 Madrid, Spain

**Keywords:** mesoporous glasses, ZnO-additions, osteostatin loading, osteosteoblast cell cultures, osteogenic effect

## Abstract

Mesoporous Bioactive Glasses (MBGs) are a family of bioceramics widely investigated for their putative clinical use as scaffolds for bone regeneration. Their outstanding textural properties allow for high bioactivity when compared with other bioactive materials. Moreover, their great pore volumes allow these glasses to be loaded with a wide range of biomolecules to stimulate new bone formation. In this study, an MBG with a composition, in mol%, of 80% SiO_2_–15% CaO–5% P_2_O_5_ (Blank, BL) was compared with two analogous glasses containing 4% and 5% of ZnO (4ZN and 5ZN) before and after impregnation with osteostatin, a C-terminal peptide from a parathyroid hormone-related protein (PTHrP_107-111_). Zn^2+^ ions were included in the glass for their bone growth stimulator properties, whereas osteostatin was added for its osteogenic properties. Glasses were characterized, and their cytocompatibility investigated, in pre-osteoblastic MC3T3-E1 cell cultures. The simultaneous additions of osteostatin and Zn^2+^ ions provoked enhanced MC3T3-E1 cell viability and a higher differentiation capacity, compared with either raw BL or MBGs supplemented only with osteostatin or Zn^2+^. These in vitro results show that osteostatin enhances the osteogenic effect of Zn^2+^-enriched glasses, suggesting the potential of this combined approach in bone tissue engineering applications.

## 1. Introduction

Bone regeneration is a natural event, but there are certain clinical situations where this physiological process is impaired. For instance, when either the bone defect to be repaired is too large or bone has lost its regenerative capacity as occurs in osteoporosis conditions. In these cases, bone regeneration needs to be stimulated by using bone tissue engineering approaches [1,2]. Such approaches use constructs formed by 3-D porous scaffolds decorated with biological signals and/or bone-forming cells. In the last decade, SiO_2_–CaO–P_2_O_5_ mesoporous bioactive glasses (MBGs) were proposed as optimum candidates for these scaffolds. These glasses exhibit bone regenerative properties and highly ordered mesoporous structures enabling binding and release bone promoting agents [1,3]. Moreover, the huge surface area and pore volumes of MBGs yield quicker in vitro responses when compared with other bioactive materials [4,5]. The behaviour of these glasses in a biological medium can be improved by incorporating bioactive metal ions in the glass network. This is the case of Zn^2+^ ions which exhibit osteogenic and angiogenic features, as well as antioxidant, cancer preventive, and antimicrobial activities [6,7,8,9,10]. In this regard, since bacterial infection [11] is an important problem after bone implant surgery [12,13], the combination of the regenerative properties of MBGs with the beneficial effects of Zn^2+^ ions has generated potential interest in bioengineering applications [14]. 

Following the bone tissue engineering principles, the bioactivity of a scaffold can be improved by loading it with osteogenic agents, such as parathyroid hormone (PTH)-related protein (PTHrP), which is emerging as an interesting promoter of bone regeneration. PTHrP contains an N-terminal 1–37 region homologous to PTH and a C-terminal PTH-unlike region containing the highly conserved 107–111 sequence osteostatin [15]. N-terminal PTHrP analogues have been shown to induce bone anabolism in rodents and humans upon systemic intermittent administration [16,17]. On the other hand, osteostatin has anti-resorptive activity [18], but also exhibits osteogenic features in vitro and in vivo [19,20,21,22,23,24]. Moreover, it has recently been shown that osteostatin coating onto various types of ceramic implants accelerates healing of critical and noncritical bone defects in the long bones of adult normal and osteoporotic rabbits and in rats [25,26,27,28,29]. Therefore, recent findings point to osteostatin as an attractive small peptide for consideration in a bone tissue engineering scenario.

In this study, the biological consequences of the concurrent inclusion of ZnO and osteostatin impregnation in MBGs were investigated. Three MBGs were synthesized, all with a basic composition of 80% SiO_2_–15% CaO–5% P_2_O_5_ (mol%), containing or not (Blank, BL) 4% or 5% ZnO, respectively (4ZN and 5ZN). These compositions were selected based on our previous studies [14], which were consistent with those reported for other glass systems showing 5% as the maximum content of ZnO enhancing osteoblast cell development without being cytotoxic [30,31,32]. MBG powders were processed as disk-shape pieces for several in vitro studies: uptake and release of osteostatin; assays in simulated body fluid (SBF); release of the inorganic ions, calcium, phosphate, and zinc from disks to the surrounding medium; and bioactivity in mouse pre-osteoblastic MC3T3-E1 cell cultures. This approach allowed us to evaluate the putative advantage of loading osteostatin onto ZnO-containing glasses to produce an optimal biomaterial for bone regeneration.

## 2. Experimental

### 2.1. Synthesis of the MBGs as Powders and Processing into Disks 

The synthesis of the MBGs was made through the EISA (Evaporation-Induced Self Assembly) method, using 4.5 g Pluronic^®^ P123 as surfactant, 85 mL ethanol (99.98%), as solvent, and 1.12 mL 0.5 N HNO_3_ as catalyst. The process was carried out for 1 h under stirring at 250 rpm, covering the flask with Parafilm^®^ to prevent the solvent evaporation. Then, the appropriate amounts of tetraethyl orthosilicate (TEOS), Ca(NO_3_)_2_·4H_2_O, triethyl phosphate (TEP) and Zn(NO_3_)_2_·6H_2_O were added as SiO_2_, CaO, P_2_O_5_ and ZnO sources, respectively (all reagents from Sigma-Aldrich, St. Louis, MO, USA). Thus, 8.9 mL TEOS were slowly added for 3 h, followed by the addition of 0.71 mL TEP for another 3 h period. Next, 1.10 g Ca(NO_3_)_2_·4H_2_O and the required amounts of Zn(NO_3_)_2_·6H_2_O depending on the designed ZnO content (0.60 g for 4ZN or 0.75 g for 5ZN) were also added. The solution was continuously stirred at 250 rpm during the synthesis process. The solution was left overnight (14 h), then it was distributed in Petri dishes (30 mL/plate), and let the ethanol to evaporate at 25 °C for 7 day. Thereafter, the resulting transparent membrane was withdrawn and heated for 6 h at 700 °C (with a heating ramp of 1 °C/min). Finally, materials were gently milled on a glass mortar to prevent deterioration of the mesoporous order and sieved through a 40 μm mesh. For the in vitro assays, the powders were conformed into disks (6 mm diameter, 2 mm height) obtained by compacting 70 mg of MBG powders with 5 MPa of uniaxial pressure.

### 2.2. Physicochemical Characterization of Samples 

The samples were characterised by CHN elemental analysis in a Macroanalyser Leco CNS-2000-I (Saint Joseph, MI, USA); Thermogravimetric and Differential Thermal analysis (TG/DTA) in the 30 °C to 900 °C interval (air flow: 100 mL/min) in a Perkin Elmer iPyris Diamond system r (Waltham, MA, USA), Fourier transformed infrared (FTIR) spectroscopy in a Thermo Scientific Nicolet iS10 apparatus (Waltham, MA, USA) equipped with a SMART Golden Gate attenuated total reflection ATR diffuse reflectance accessory; Small-Angle X-ray diffraction, SA-XRD, in a X'pert-MPD system (Eindhoven, The Netherlands) equipped with Cu Kα radiation in the 0.6 to 8° 2θ range and Transmission Electron Microscopy (TEM), in a JEM-2100 JEOL microscope operating at 200 kV (Tokyo, Japan). Samples were ultrasonically dispersed in n-butanol and deposited in a copper grid coated with a holed polyvinyl-formaldehyde layer for TEM analysis. 

Moreover, samples were characterised by nitrogen adsorption and solid-state nuclear magnetic resonance (NMR). Nitrogen porosimetry was performed in a Micromeritics ASAP 2020 (Norcross, GA, USA). Samples were previously degassed 24 h at 120 °C under vacuum. The surface areas were calculated by the Brunauer-Emmett-Teller (BET) method [33], and the pore size distributions by the Barret–Joyner–Halenda (BJH) method [34]. Surface functionalization was studied by solid state single pulse magic angle spinning nuclear magnetic resonance (SP MAS NMR). The ^29^Si and ^31^P spectra were obtained on a Bruker Avance AV-400WB spectrometer (Karlsruhe, Germany) equipped with a solid state probe using a 4 mm zirconia rotor and spun at 10 kHz for ^29^Si and 6 kHz in the case of ^31^P. Spectrometer frequencies were set at 79.49 and 161.97 MHz for ^29^Si and ^31^P, respectively. Chemical shift values were referenced to tetramethylsilane (TMS) for ^29^Si and H_3_PO_4_^31^P. The time period between accumulations were 5 and 4 s for ^29^Si and ^31^P, respectively, and the number of scans was 10,000.

### 2.3. In Vitro Studies

In vitro tests were carried out in MBGs disks sterilized for 20 min under UV radiation (10 min/face) in a laminar flux cabinet. The disks maintained their stability without crumbling even for soaking times as long as 21 days in the assays performed in SBF. 

#### 2.3.1. Adsorption and Release of Osteostatin 

For the adsorption assay, the disks in 24-well plates were incubated with 1 mL of phosphate-buffered saline (PBS), pH 7.4, containing or not (control) 100 nM osteostatin. Samples were left under stirring at 400 rpm, at 4 °C. Osteostatin adsorption in each type of tested MBG after 24 h was calculated based on the peptide removed from the liquid medium; whereas osteostatin release was measured by soaking the peptide-loaded disks for different times (1, 2, 24, 48, 72, and 96 h) in PBS also under stirring, at 37 °C. The amount of osteostatin in PBS medium was measured by UV spectrometry at 280 nm using a NanoDrop ND-2000 (NanoDrop Technologies, Thermo Fisher Scientific, Wilmington, DE, USA). 

#### 2.3.2. Assays in SBF 

In vitro bioactivity tests were carried out by soaking the disks for 6 h, 24 h, 3 days, 7 days, 14 days, and 21 days in SBF, pH 7.4, at 37 °C [35]. SBF was previously filtered through a 0.22 µm filter to prevent bacterial contamination. The disks were placed in polyethylene flasks containing 13 mL of SBF, according to the equation Vs = Sa/10 (being Vs the SBF volume in mL and Sa the external surface area of the disks in mm^2^). Inside the SBF, the disks were located in a vertical position by including them in “baskets” fabricated with platinum wire. Two replicas by material and time and a control with only SBF were included.

Before and after the assays, disks were characterized by wide angle X-ray diffraction (XRD, 2θ from 10–70) in an X’Pert-MPD (Philips) system, FTIR spectroscopy in a Thermo Scientific Nicolet iS10 (KBr pellet method), and SEM in a JSM-6400 (JEOL) microscope (Tokyo, Japan) coupled with an EDX spectroscopy system (Oxford Instruments, Abingdom, UK). Moreover, changes in Ca^2+^ concentration and pH of the liquid medium were assessed with an ILyte^®^ electrode ion selective system (Diamond Diagnostics, Holliston, MA, USA). An in vitro bioactive behaviour in SBF is generally identified by the deposition on the material surface of amorphous calcium phosphate (ACP) layer that later on crystallized as hydroxycarbonate apatite (HCA) nanocrystals analogous to those in bone [36,37]. 

#### 2.3.3. Ions Release from Disks

The release of ions was investigated by soaking the MBG disks in 2 mL of Dulbecco’s modified Eagle medium (DMEM) (Sigma-Aldrich. St. Louis, MO, USA) supplemented with 10% fetal bovine serum (FBS) and antibiotics (100 U mL^−1^ penicillin, 100 mg mL^−1^ streptomycin) (usually called “complete medium”) at 37 °C for different times between 24 h and 5 days. For each disk sample, the cumulative amounts of Ca, P and Zn released to the complete medium was determined by inductively coupled plasma/optical spectrometry (ICP/OES) using an OPTIMA 3300 DV device (Perkin Elmer). The concentration of each ion was determined from three replicates on the same solution split into two independent experiments.

#### 2.3.4. Culture Cell Studies

Cell culture experiments were performed using the mouse pre-osteoblastic MC3T3-E1 cell line (subclone 4, CRL-2593; ATCC, Mannassas, VI) [25,27]. The different disks tested were placed into 6 and 24-well plates before cell seeding at 20,000 cells/cm^2^ in 2 mL of α-minimum essential medium containing 10% FBS, 50 μg/mL ascorbic acid, 10 mM β-glycerol-2-phosphate, and 1% penicillin–streptomycin at 37 °C in a humidified atmosphere of 5% CO_2_, and incubated for different times between 1 and 13 days. As controls, wells without disks were used. The medium was replaced every other day.

Cell numbers were determined using the CellTiter 96^®^ AQueous Assay (Promega, Madison, WI, USA), a colorimetric method for determining the number of living cells in the culture. Cells were cultured without (control) or with the tested disks for 10 h (only measured on the disks surface) or for 2 and 5 days (measured in both the disks and well surface). Next, 40 μL of CellTiter 96^®^AQueous One Solution Reagent [containing 3-(4,5-dimethythizol-2-yl)-5-(3-carboxymethoxyphenyl)-2-(4-sulfophenyl)-2*H*-tetrazolium salt (MTS) and an electron coupling reagent (phenazine ethosulfate) that allows its combination with MTS to form a stable solution for 4 h] was added to each well (200 μL) in contact with the cells. The quantity of formazan product as measured by the amount of 490 nm absorbance (in a Unicam UV-500 spectrophotometer) is directly proportional to the number of living cells in culture. In addition, in some cell cultures, at 2 days, cells were trypsinized and counted in a hemocytometer to determine cell death by Trypan blue exclusion.

Alkaline phosphatase (ALP) activity was measured in MC3T3-E1 cell extracts obtained with 0.1% Triton X-100 at day 5 of culture, using p-nitrophenylphosphate as substrate, as previously described [38]. ALP activity was normalized to the cell protein content, determined by the Bradford’s method using bovine serum albumin as standard.

Matrix mineralization was measured in MC3T3-E1 cell cultures by alizarin red staining, as described [26]. After incubation with the different disks for 12 days, cells were washed with PBS, and fixed with 75% ethanol for 1 h at room temperature. Cell cultures were stained with 40 mM alizarin red (pH 4.2) for 10–30 min at room temperature. Then, cells were washed with distilled water, and the stain was dissolved with 10% cetylpyridinum chloride in 10 mM PBS and measuring absorbance at 620 nm in a Unicam UV-500 spectrophotometer (ThermoSpectronic, Cambridge, UK).

Total RNA was isolated from MC3T3-E1 cells by a standard procedure (Trizol, Invitrogen, Groningen, The Netherlands), and gene expression was analysed by real-time PCR using a QuantStudio 5 Real-Time PCR System (Thermo Fisher Scientific, Wilmington, DE, USA). Real-time PCR was done using mouse-specific primers and TaqMan^MGB^ probe for Runx2 (Assay-by-Design^SM^, Applied Biosystems, CA, USA). The mRNA copy numbers were calculated for each sample by using the cycle threshold (C_t_) value. Glyceraldehyde 3-phosphate dehydrogenase (GAPDH) rRNA (a housekeeping gene) was amplified in parallel with Runx2. The relative gene expression was represented by 2^−ΔΔCt^, where ΔΔCt = ΔCt_target gene_ − ΔCt_GAPDH_. The fold change for the treatment was defined as the relative expression compared with control, calculated as 2^−ΔΔCt^, where ΔΔCt = ΔC_treatment_ − ΔC_control_ [21]. Runx2 (Mm00501578_m1; NM_001146038.2). GAPDH (Mm99999915_g1; NM_001289726.1).

Cell morphology was studied in disks using an Eclipse TS100 inverted optical microscope (Nikon) (Amsterdam, The Netherlands) after 24 h. Fluorescence microscopy was also carried out for the observation of attached cells onto the disks. After samples were fixed and permeabilized, they were incubated with Atto 565-conjugated phalloidin (dilution 1:40, Molecular Probes, Sigma-Aldrich, St. Louis, MO, USA), which stains actin filaments. Then, samples were washed with PBS and the cell nuclei were stained with l M diamino-20-phenylindole in PBS (DAPI) (Molecular Probes). Fluorescence microscopy was performed with an EVOS FL Cell Imaging System (Waltham, MA, USA) equipped with tree Led Lights Cubes (kEX (nm); kEM (nm): DAPI (357/44; 447/60), RFP (531/40; 593/40) from AMG (Advance Microscopy Group, Bothell, WA, USA).

#### 2.3.5. Statistical Analysis

Results are expressed as mean ± SEM (SEM: standard error of mean). Statistical evaluation was carried out with nonparametric Kruskal-Wallis test and post-hoc Dunn´s test, when appropriate. A value of *p* < 0.05 was considered significant.

## 3. Results and Discussion

### 3.1. Glass Powders Characterization

Prior to obtaining t, he disks that were used for the in vitro tests, the MBG powders were characterized by several experimental techniques. CHN elemental analysis, TG/DTA, and FTIR spectroscopy showed the successful synthesis of glasses confirming the entire removal of surfactant and nitrate groups coming from Ca^2+^ and Zn^2+^ sources, and the MBG stabilization under ambient conditions after the last step of synthesis, namely the treatment at 700 °C. In addition, MBGs powders were characterised by SA-XRD and TEM, to assess if they exhibited ordered mesoporosity, and by nitrogen adsorption to determine their textural properties, i.e., specific surface area and porosity. 

**Figure 1:**
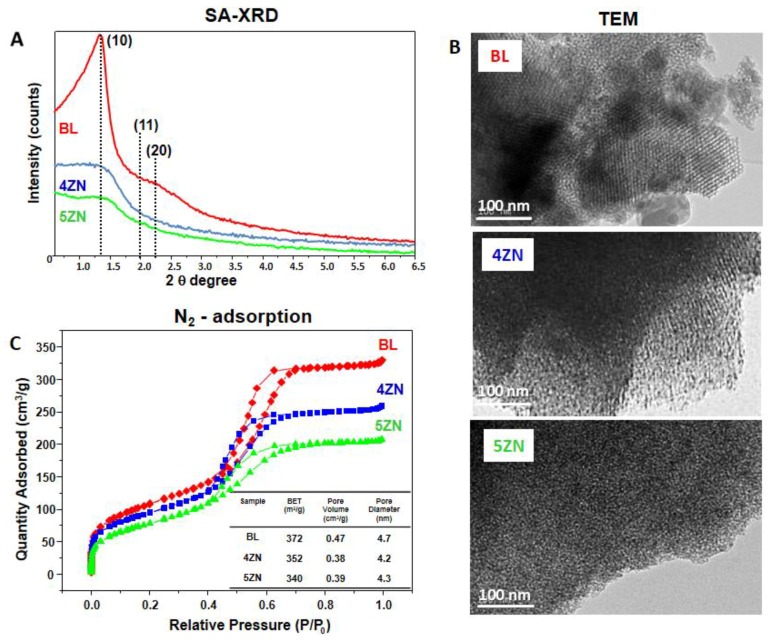
Physicochemical characterization of Zn-free (BL) and Zn-substituted (4ZN and 5ZN) MBG powders by: (**A**) SA-XRD; (**B**) TEM; and (**C**) N_2_ adsorption. Inset table: calculated textural properties, i.e., specific surface area (S_BET_), volume of pores (V_P_), and pore diameter (D_P_).

Figure 1A shows the SA-XRD patterns of BL, 4ZN, and 5ZN powders. As is observed, the BL pattern exhibits a sharp diffraction maximum at 1.3, indicative of mesoporous order, and a shoulder at around 2.0 in 2θ. According to our previous studies, the sharp maximum was assigned to the (10) reflection of a 2-D hexagonal phase formed by the mesopores arrangement and the shoulder to the low intensity (11) and (20) reflections of this phase [39]. In contrast, in the 4ZN and 5ZN patterns, only a diffuse maximum and shoulders at about 1.3 in 2θ were observed. This type of pattern is generally present in samples exhibiting worm-like order [40].

Figure 1B shows the high resolution TEM images of the MBG powders. BL and 4ZN images mainly show ordered areas confirming the presence of a mesoporous ordered structure. In addition, in these samples minority regions with disordered worm-like structures are present. In the TEM image of 5ZN, most of the observed areas exhibited worm-like order. Thus, TEM results confirmed those obtained by SA-XRD, demonstrating that the order of mesopores decreased with the presence of Zn^2+^ ions in the glass network. 

To assess whether this decrease in the mesoporous order by Zn^2+^ ions was accompanied by a significant variation in the textural parameters, the MBG powders were characterized by nitrogen adsorption. As observed in Figure 1C, the isotherms of the three samples were type IV, characteristic of mesoporous materials. Moreover, the curves exhibit a type H1 cycle of hysteresis, indicative of the presence of cylindrical pores opened at both ends. Thus, BL, 4ZN, and 5ZN exhibited analogous features in terms of the type and shape of the pores present. The aforementioned textural properties of these glasses were then calculated from the isotherms. As seen in Figure 1C, inset, only moderate decreases took place in the textural properties as consequence of the inclusion of Zn^2+^ ions in the glass. Thus, the specific surface area of 372 m^2^/g of BL slightly decreased to 362 and 340 m^2^/g in 4ZN and 5ZN, respectively. Furthermore, the pore volume also experienced a moderate decrease from 0.47 to 0.38 and 0.39 cm^3^/g, and the average pore diameter from 4.7 to 4.2 and 4.3 nm, respectively, in Zn-containing glasses.

**Figure 2:**
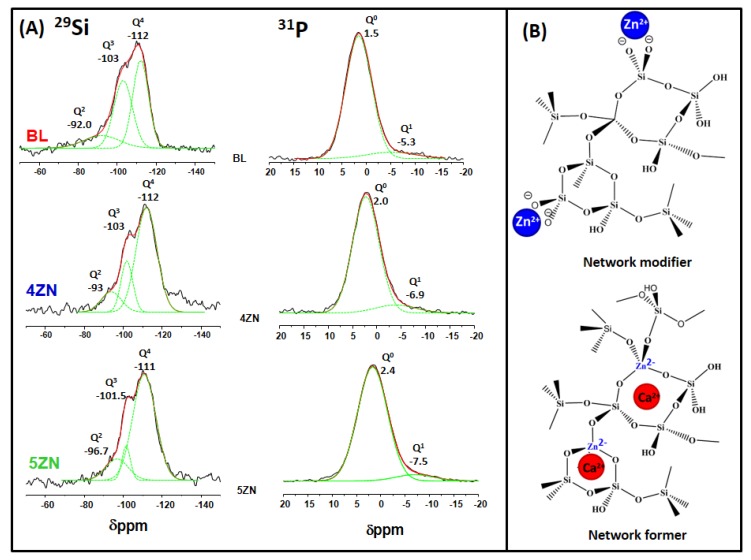
(**A**) Solid-state ^29^Si single-pulse and ^31^P single-pulse MAS-NMR spectra of BL, 4ZN and 5ZN. Q^n^ unit areas were calculated by Gaussian line-shape deconvolution and displayed in green; (**B**) Schematic view of Q^2^ Zn and Q^4^ Zn assignments from ^29^Si MAS NMR.

^29^Si and ^31^P solid state MAS NMR measurements were carried out to investigate the environments of the network formers and network modifiers species at atomic level in the MBGs (Figure 2). The NMR analysis will be related later with the release of Zn^2+^ ions in the in vitro assays with cells. In the Figure, Q^2^, Q^3^, and Q^4^ represent, respectively, the silicon atoms (denoted Si*) in (NBO)_2_Si*–(OSi)_2_, (NBO)Si*–(OSi)_3_, and Si*(OSi)_4_ (NBO = nonbonding oxygen) [41], whereas Q^0^ and Q^1^, represent respectively the phosphorus atoms (denoted P*) in the PO_4_^3−^species, (NBO)_3_P*–(OP) and (NBO)_2_-P*–(OP)_2_ (NBO relative to another P atom). The chemical shifts, de-convoluted peak areas, and silica network connectivity <Q^n^> for each glass composition are collected in Table 1. 

In the ^29^Si NMR spectra, the signals at −110 to −112 ppm region were assigned to Q^4^; at −101 to −103 ppm to Q^3^; and at −92 ppm to −96 ppm to Q^2^. The BL sample was characterized by a high percentage of Q^4^ and Q^3^ species, and the network connectivity, <Q^n^>, calculated for this sample was 3.57. This value is lower than reported for a sol-gel glass with identical composition that was 3.75 [40]. The relatively low values of <Q^n^> in MBGs is one of their features that can explain the quick in vitro bioactive response of this family of glasses. 

As is observed in the Table, the inclusion of 5.0% ZnO produced an increase of <Q^n^>, 3.67, compared with BL whereas in 4ZN a slight decrease, 3.55, was observed. These results were explained considering that when a 4.0% of ZnO was added, Zn^2+^ ions behaved as network formers with tetrahedral coordination [ZnO_4_] which exhibit negative charge (2^−^). These tetrahedra attract Ca^2+^ ions that accordingly behave as charge compensators instead of as network modifiers (Figure 2B). Regarding 5ZN, the higher percentage of Q^4^ species in this sample indicates a decrease in the NBO which supposes a higher contribution of Zn^2+^ as the network former compared with 4ZN, explaining the highest value of <Q^n^> for 5ZN. However, the amounts of ZnO were not high enough to increase substantially the depolymerisation of network in 4ZN and 5ZN with respect to BL, explaining the similar values of <Q^n^> obtained for the three samples (Table 1) that agree with previously reported for BL and 4ZN [14]. The increasing FWHM was due to a larger distribution of isotropic values of the chemical shift, with is caused by a decreasing short-range order of the framework structure [42]. The tetrahedral symmetry of the Q^4^ units in BL sample respect to 4ZN and 5ZN samples indicates an isotropic structure when no zinc was added to the MBGs. In addition, the crystallinity of Q^3^ and Q^2^ were slightly greater when zinc was present in the samples. 

On the other hand, the ^31^P NMR spectra show a maximum of ≈2 ppm assigned at the Q^0^ environment of amorphous orthophosphate (Figure 2A) and a second weak signal from −5.2 ppm to −7.5 ppm when the ZnO % in MBGs increases [43]. This resonance fell in the range of Q^1^ tetrahedra and can be assigned to P–O–Si environments as previously reported [44,45]. Thus, P was mainly present as orthophosphate units but Zn inclusion caused a slight decrease of Q^1^ units percentage, and its chemical shift pass from −5.2 ppm for BL to −7.5 ppm for 5ZN, suggesting a partial conversion of P–O–Si units into P–O–Zn units due to Zn^2+^ ion acting as a network former with more anisotropic structure than Q^1^ of the BL sample. The formation of P–O–Zn was proposed for bioactive melt glasses where a shift towards lower ppm was detected when the ZnO % in the glass increased [46].

In summary, the characterizations of the MBGs powders has shown the decrease of the order of mesopores when ZnO in glasses increased. However, the textural properties of ZnO-containing MBGs remained similar to un-doped MBG (BL) with values of surface area and porosity higher than conventional sol-gel glasses [47]. Moreover, the NMR results allowed for understanding the release of Zn^2+^ ions during the in vitro assays with cells.

### 3.2. Textural Properties of MBG Disks

**Figure 3:**
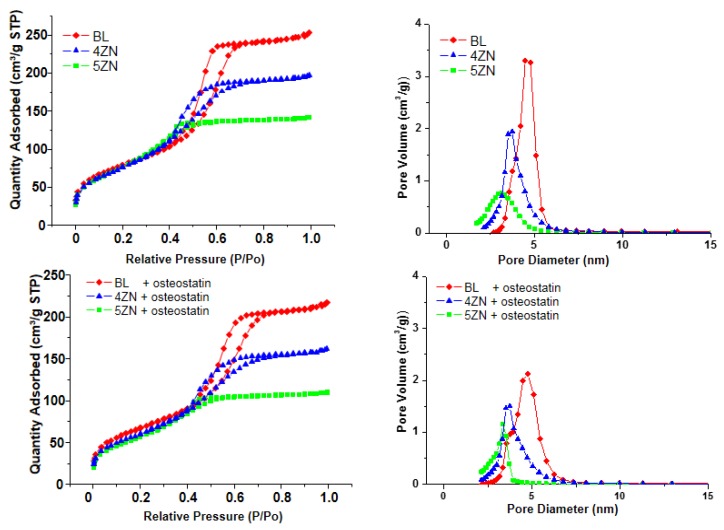
N_2_ adsorption-desorption isotherms (left) and pore size distribution (right) of BL, 4ZN, and 5ZN disks before (upper panels) and after (lower panels) being loaded with osteostatin.

As previously mentioned, the disks used for the in vitro tests were obtained by compacting the powders at 5 MPa, then it was necessary to characterize the MBG disks by nitrogen adsorption to evaluate the textural properties after the processing. As observed in the top-left of Figure 3, the isotherms showed identical features to the MBG powders shown in Figure 1C. Moreover, at the top-right of Figure 3, the corresponding pore size distributions are shown.

Table 2 allows for the comparison of the textural properties of MBG powders and disks. As it is observed, the disks exhibited moderate decreases of the textural properties compared with the corresponding powders. Thus, S_BET_ values, between 372 and 340 m^2^/g in powders, decreased to values in the range 287–280 m^2^/g in the disks, and the pore volume decreased from 0.47–0.38 cm^3^/g to 0.38–0.22 cm^3^/g. Therefore, textural propertied of disks remained high enough to host osteostatin molecules. The textural properties of disks after being loaded with osteostatin are also included in Table 2. As is observed, additional decreases of the specific surface area and pore volume were detected, confirming the loading of the osteostatin into the MBGs. Finally, the composition of disks was determined by EDX obtaining the values shown at the right of the Table 2. These values showed a good agreement with the nominal composition of the glasses included between brackets in the table.

### 3.3. Uptake and Release of Osteostatin

**Figure 4:**
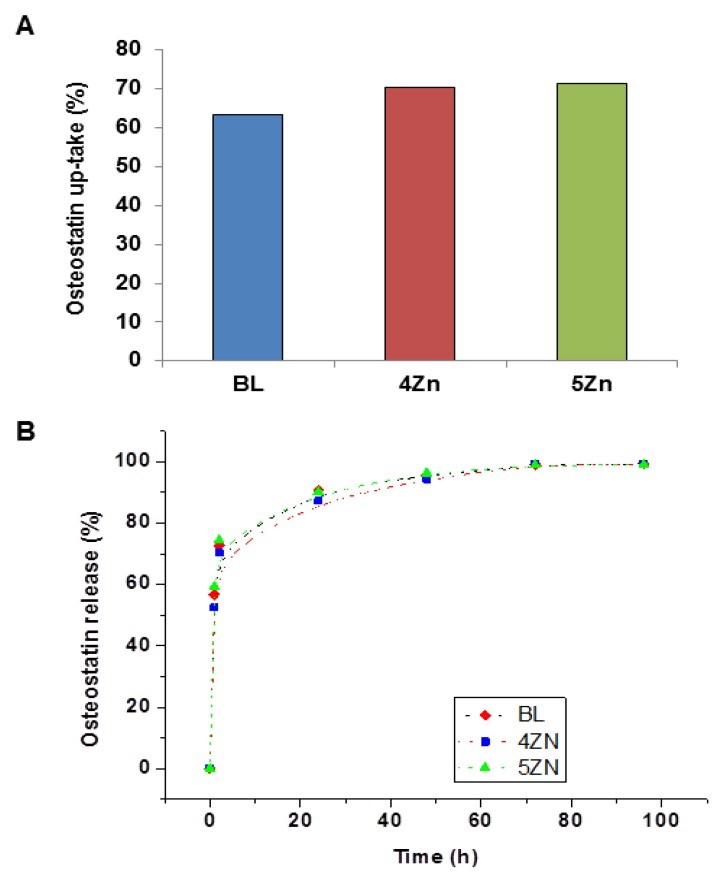
Osteostatin uptake at 24 h (**A**), and its release profiles measured at different times for BL, 4ZN, and 5ZN disks (**B**). Points tracing the curve are the means of three experiments.

After soaking the MBG disks in a 100 nM solution of osteostatin in PBS for 24 h, the mean uptake of the peptide was 63% (BL), 70% (4ZN), and 71% (5ZN) (Figure 4A), equivalent to 0.8, 0.9, and 0.95 µg/g per disk, respectively. 

On the other hand, the osteostatin released from the loaded disks to the medium after 1 h was 73% (BL), 67% (4ZN), and 68% (5ZN). After 24 h, it was 95% (BL) or 90% (4ZN and 5 ZN) and it was virtually 100% for the three MGBs at 96 h (Figure 4B). It is pertinent to mention here that minimum amounts of this peptide (even in the sub-nM range) were efficient to induce osteogenic activity [26,27,28].

As previously told, the effect of loading osteostatin in the textural properties of disks was determined. Figure 3 includes the N_2_ adsorption isotherms (bottom-left) and pore size distributions (bottom-right) of osteostatin-loaded disks. As is observed in Table 2, a slight decrease in the surface area and porosity was observed in the MBG disks as a consequence of osteostatin loading. These results suggest that a part of the peptide loading took place inside the pores, but without affecting the ordering of the mesopore channels.

If we assume that the release mechanism of osteostatin was diffusion through the mesopores and considering the low solubility of the glasses at the medium pH (7.4), the peptide release could be described by a deviation from the theoretical first-order behaviour of the Noyes–Whitney equations as described by Equation (1) [48,49]:

W_t_/W_0_ =A(1 − exp^k^_1_^·t^) (1)

where W_t_ stands for the peptide mass released at time t; W_0_ represents the maximum initial mass of the peptide inside the pores; A is the maximum amount of peptide released; and k_1_ is the release rate constant, which is independent of peptide concentration and gives information about the solvent accessibility and the diffusion coefficient through mesoporous channels.

This model was successfully applied for the release of different drugs from insoluble mesoporous matrices with a similar structure [48]. According to this model, peptide release is faster within the first 24 h, reaching a stationary phase after 48 h. This deviation could be due to several factors, such as the peptide volume, the distortion of the mesopore channels, and/or the release of peptide molecules adsorbed on the external surface of the matrices.

This divergence has been dealt with by the introduction of an empirical non-ideality factor δ in Equation (2) [50]:

W_t_/W_0_ = A(1 − exp^k^_1_^·t^)^δ^ (2)

The values of this non-ideality factor δ range from 1–0 for materials that either follow first-order kinetics or initially release the peptide molecules located on the external surface of the matrices. The obtained data were fitted using this semi-empirical first-order model, and the release parameters are shown in Table 3. According to this model, δ gives an idea of the degree of fidelity of this approximation. In all tested MBG matrices, the δ value was low and similar, indicating that a relatively large percentage of osteostatin molecules released from the external surface of the MBGs. Moreover, the percentage of osteostatin released was maximal, indicating virtually no osteostatin long retention by all these MBGs.

### 3.4. In Vitro Bioactivity Assay

The different MBG disks before and after soaking in SBF for different times were characterized by FTIR. This is a very sensible technique for detecting the formation of amorphous calcium phosphate (ACP) and HCA by evaluating the region of the spectra at around 600 cm^−1^. The presence of a band in this region is characteristic of ACP, and the split of this band in bands at 560 and 603 cm^−1^ is indicative of phosphate in a crystalline environment like the one in nano-HCA [51]. 

**Figure 5:**
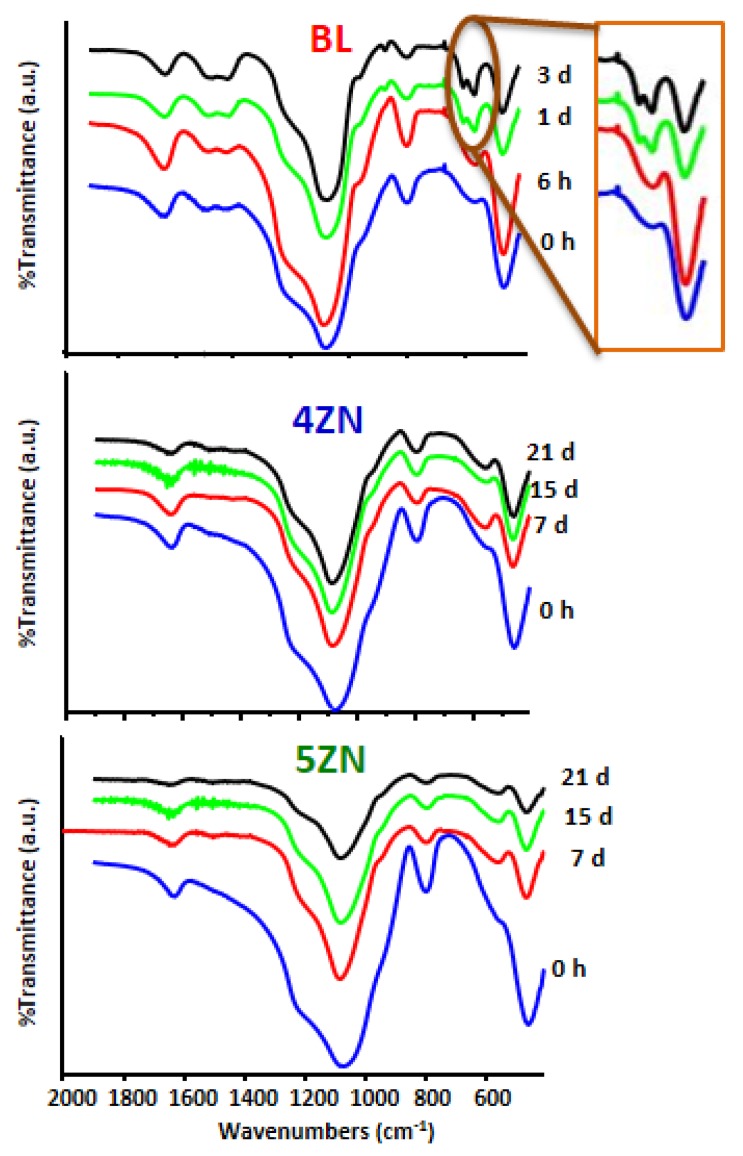
FTIR spectra of BL, 4ZN, and 5ZN after different times in SBF with 100 nM of osteostatin. The ellipse highlights the bands of phosphate in a crystalline environment.

As observed in Figure 5, the behaviour of BL in SBF was different from that of 4ZN and 5ZN. Thus, for the Zn-free MBG, the band at 600 cm^−1^ was visible at 6 h of incubation, and the bands of HCA were already detected at 1 day. For longer times, like 3 days, the FTIR spectra did not suffer additional changes. However, for 4ZN and 5ZN only the band of ACP was observed after 7 days of treatment and the two bands of HCA were not detected even for soaking days as long as 21 days. These results had already been described for bioactive MBGs to which the ZnO additions impeded the formation of HCA in regular SBF (osteostatin-free) [39]. This result was thought to be a consequence of the initial formation of amorphous calcium zinc phosphate, unable to crystallize as HCA. However, in the present study we demonstrated that, although the impregnation of MBGs with 100 nM osteostatin solution exerted a remarkable effect in the MBGs’ behaviour in the presence of cells (see the next sections), osteostatin was not found to affect the in vitro formation of ACP or HCA on the tested glasses in SBF.

**Figure 6:**
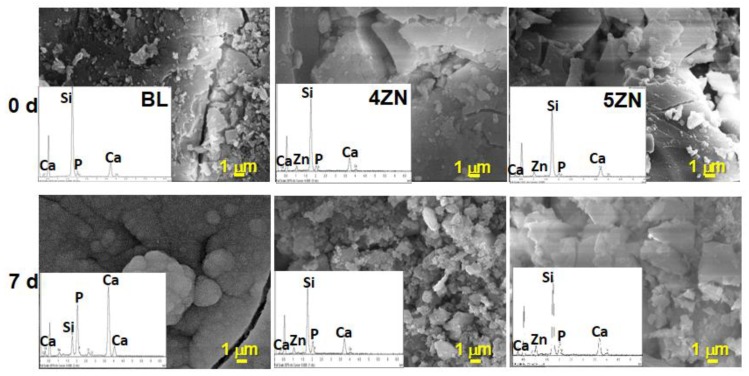
SEM micrographs and EDX spectra of BL, 4ZN, and 5ZN MBG disks, before and after being soaked for 7 days in SBF.

SEM analysis confirmed the FTIR results. Thus, Figure 6 shows the SEM micrographs of BL, 4ZN, and 5ZN before and after being soaked for 7 days in SBF. As is observed, after this time only BL appeared coated by a layer of spherical particles with the characteristic morphology of bone-like HCA. This morphology was developed from the initially formed flocculent shape of ACP. In contrast, for 4ZN and 5ZN disks, no HCA layer was found after 7 days immersed in SBF, although a new material was observed on the 4ZN surface. However, the high intensity of the calcium and phosphorous peaks in the EDX spectra of BL, compared to the other samples, supports the interpretation that HCA preferentially showed up on the BL sample. These results were analogous to those reported for Zn-containing MBGs performed in pure SBF showing the inhibitory effect of Zn^2+^ ions in the HCA crystallization [39]. In the present study, we obtained the identical results demonstrating the null effect of osteostatin additions in the assays in the acellular SBF, in spite of the important effect that exerts in the presence of cells as it will be described in the following sections.

### 3.5. Degradability of Disks in Complete Medium

**Figure 7:**
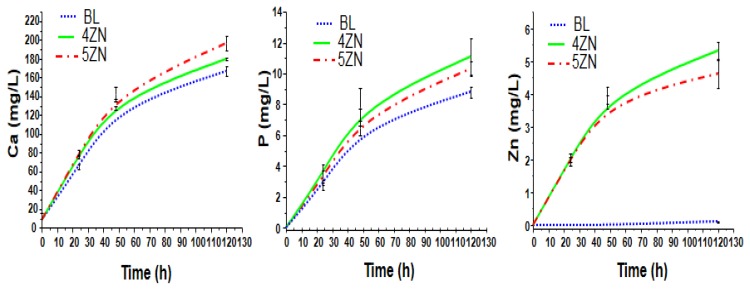
Evolution of cumulative calcium, phosphorus, and zinc content of osteostatin-loaded disks as a function of time in a complete medium.

To better understand the MBGs cytocompatibility, the release of calcium, phosphorus, and zinc ions from osteostatin-loaded disks after being soaked for 130 h in a complete medium were measured. As it is shown in Figure 7, in BL, calcium and phosphorous concentrations in solution were slightly less than in 4ZN and 5ZN, which can be explained by the HCA layer formed on BL as it was mentioned in the previous section. This variation fit well with the formation of ACP during this interval. Moreover, the Zn concentration in Zn-substituted scaffolds increased until day 5 in both 4ZN and 5ZN disks, reaching a value of 4.6 and 5.3 ppm, respectively; this is consistent with the slightly higher network polymerization of 5ZN material compared to 4ZN, thus releasing less amount of Zn^2+^ to the medium. 

### 3.6. Cell Culture Studies 

**Figure 8:**
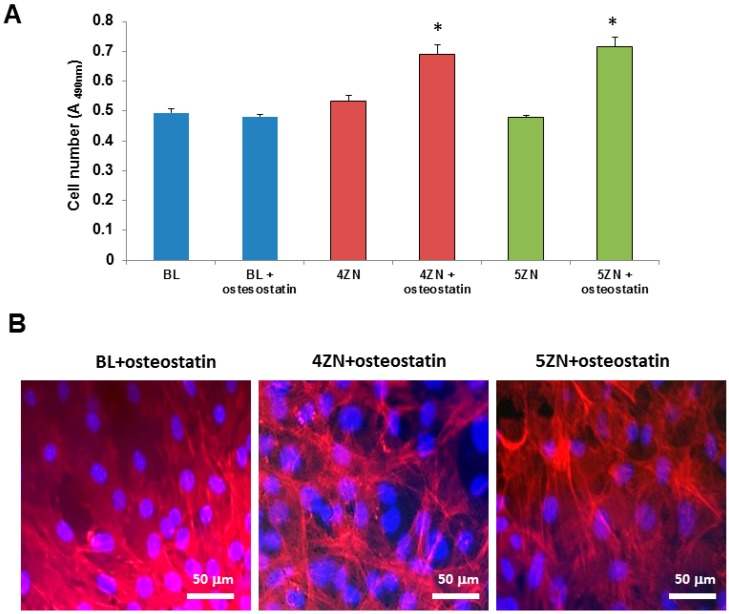
MC3T3-E1 cell number onto BL, 4ZN, and 5ZN disks measured at 10 h of cell culture. (**A**) Results are means ± SEM of three measurements in triplicate (* *p* < 0.05) vs. the corresponding unloaded disks. Absorbance was measured at 490 nm, directly proportional to the number of living adherent cells. Cell morphology evaluation performed by light microscopy onto BL, 4ZN, and 5ZN disks at day 5 of cell culture; (**B**) Cells were stained with DAPI (blue) for the visualization of the cell nuclei and phalloidin-565 (red) for the visualization of cytoplasmic F-actin filaments.

**Figure 9:**
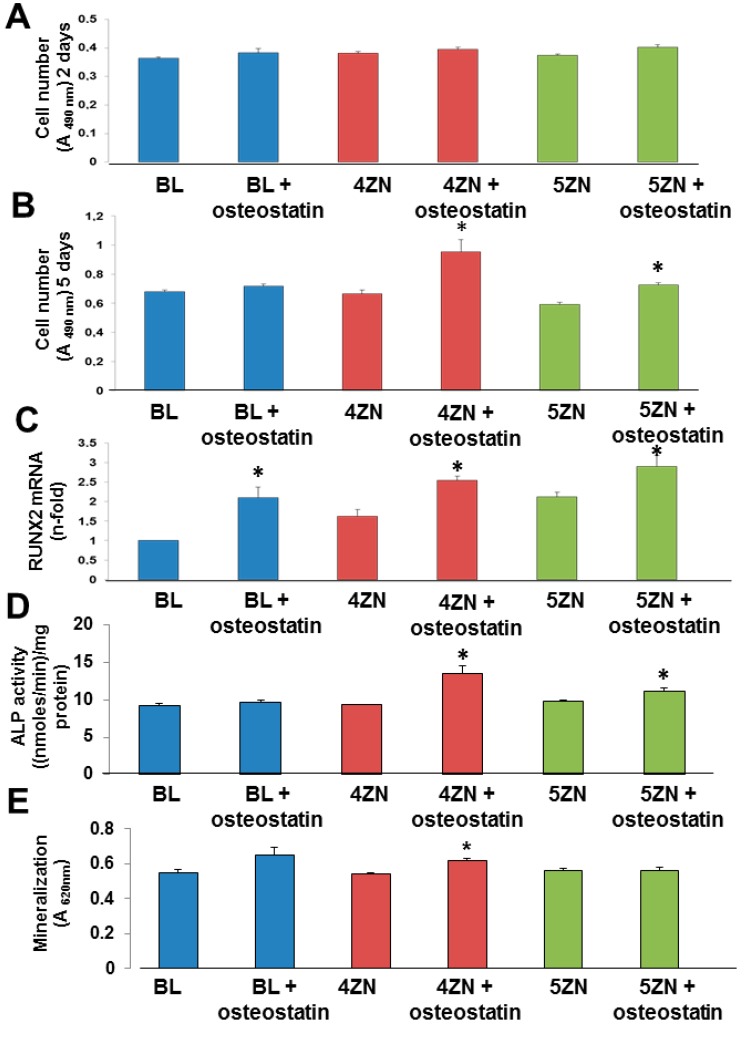
MC3T3-E1 cell number measured by CellTiter 96^®^ AQueous assay in the presence of BL, 4ZN, and 5ZN disks, loaded or not with 100 nM osteostatin, after 2 days (**A**) and 5 days (**B**) of culture. Absorbance was measured at 490 nm, directly proportional to the number of living cells. Runx2 mRNA levels measured by real-time PCR (**C**), ALP activity (**D**), and matrix mineralization measured by Alizarin red staining (**E**) in MC3T3-E1 cells in the presence of these materials at 5 days (**C**,**D**) and 12 days (**E**) of culture. For mineralization studies, absorbance was measured at 620 nm. Results are means ± SEM of three measurements in triplicate (* *p* < 0.05) vs. corresponding unloaded disks.

We next examined and compared the osteogenic activity conferred by Zn^2+^ and osteostatin to these MBGs using MC3T3-E1 pre-osteoblastic cell cultures. We first showed that cell numbers onto the disk’s surface at 10 h of culture was increased in both 4ZN and 5ZN materials loaded with osteostatin (Figure 8A). At day 5 of cell culture, the cellular morphology was not modified by any tested material (Figure 8B). Consistent with this result, although Zn^2+^ in these MBGs failed to affect cell number, the presence of osteostatin in both 4ZN and 5ZN materials increased this parameter significantly after 5 days of culture (Figure 9A,B). This pattern of bioactivity matched the amount of Zn^2+^ released to the surrounding medium (Figure 7). Thus, although osteostatin loaded into BL disks exhibited a tendency (but not significant) to increase cell viability (Figure 9A,B), this was only clearly displayed with the peptide-coated 4ZN and 5ZN glasses. None of the tested materials induced significant cell death (about 1%), assessed by Trypan blue exclusion, in these cell cultures (data not shown).

We next evaluated the capacity of these MBGs to affect osteoblastic cell differentiation. The expression of the early osteoblast differentiation marker Runx2 was increased by the presence of osteostatin in each type of MBG disks at day 5 of MC3T3-E1 cell culture (Figure 9C). Moreover, while 4ZN or 5ZN disks had minimal effect on ALP activity or matrix mineralization in these cells, coating with osteostatin increased these differentiation parameters, mainly in the 4ZN material (Figure 9D,E). 

In summary, in vitro studies demonstrated that MBG disks could be loaded with osteostatin, which was mostly released in 24 h. Osteostatin improved the cytocompatibility of Zn-containing MBGs by enhancing osteoblastic proliferation and differentiation without affecting their HCA formation capability. Further studies in vitro and in vivo are needed to elucidate the optimum material, using porous 3-D scaffolds of these MBGs, for bone tissue engineering applications. 

## 4. Conclusions

The results obtained in this study provide a novel and interesting insight in the field of bioactive glasses for bone regeneration. MBG disks containing 4 or 5% ZnO and decorated with osteostatin were shown to improve osteoblastic cell number as well as osteoblast differentiation capacity. For the first time, osteostatin was demonstrated to enhance the in vitro osteogenic capacity of Zn^2+^-enriched materials, suggesting the potential of this approach in bone tissue engineering applications.

## Figures and Tables

**Table 1:** Chemical shifts (CS) and relative peak areas of MBGs obtained by ^29^Si and ^31^P NMR. Areas of the Q^n^ units were calculated by Gaussian deconvolution, the relative populations were expressed as % and the full width at half maximum, FWHM, was also included.

Sample	^29^Si										^31^P					
	Q^4^			Q^3^			Q^2^				Q^0^			Q^1^		
	CS ppm	Area (%)	FWHM ppm	CS ppm	Area (%)	FWHM ppm	CS ppm	Area (%)	FWHM ppm	<Q^n^>	CS ppm	Area (%)	FWHM ppm	CS ppm	Area (%)	FWHM ppm
BL	−112	61.7	8.04	−103	33.8	9.4	−92	4.4	18.5	3.57	1.5	91.2	5.7	−5.3	8.8	13.0
4ZN	−112	68	10.6	−102	18.9	6.0	−94	12.9	10.3	3.55	2.0	93.7	7.7	−6.9	6.3	6.1
5ZN	−110	75.7	11.8	−103	15.4	4.3	−93.5	8.92	11.6	3.67	2.4	92.9	5.8	−7.5	7.0	7.4

**Table 2:** Textural properties of the glasses as powders, disks, and disks loaded with osteostatin. (S_BET_: specific surface area; V_T_: pore volume; D_P_: pore diameter). On the right, experimental compositions of samples determined by EDX and nominal compositions indicated between brackets are given.

	Powders			Disks			Disks + Osteostatin			Composition (EDX) Atomic %			
	S_BET_ (m^2^/g)	V_T_ (m^3^/g)	D_P_ (nm)	S_BET_ (m^2^/g)	V_T_ (m^3^/g)	D_P_ (nm)	S_BET_ (m^2^/g)	V_T_ (m^3^/g)	D_P_ (nm)	SiO_2_	CaO	P_2_O_5_	ZnO
BL	372	0.47	4.7	287	0.38	4.7	244	0.32	4.6	77.0 (80)	6.1 (5)	16.8 (15)	--
4ZN	352	0.38	4.2	280	0.30	3.7	221	0.24	3.6	74.3 (77)	7.0 (4.8)	14.3 (14.4)	4.2 (4)
5ZN	340	0.39	4.3	285	0.22	3.3	208	0.17	3.1	68.1 (76)	9.2 (4.8)	17.3 (14.3)	5.3 (5)

**Table 3:** Kinetic parameters of osteostatin release from BL, 4ZN, and 5ZN materials. (w_0_: initial loaded mass: μg ost/g MBG; A: maximum amount of peptide released; k_1_: release rate constant; δ: kinetic non-ideality factor; R: goodness of fit.

	W_0_ (g/g)	A (%)	k_1_(*10^3^) (h^−1^)		R
BL	0.80	99.3 ± 3.3	37.7 ± 3	0.14 ± 0.04	0.996
4ZN	0.90	99.7 ± 3.9	31.5 ± 3	0.13 ± 0.04	0.998
5ZN	0.95	99.6 ± 3.9	31.5 ± 4	0.12 ± 0.07	0.996
